# Identification of a Blood-Based Protein Biomarker Panel for Lung Cancer Detection

**DOI:** 10.3390/cancers12061629

**Published:** 2020-06-19

**Authors:** Victoria El-Khoury, Anna Schritz, Sang-Yoon Kim, Antoine Lesur, Katriina Sertamo, François Bernardin, Konstantinos Petritis, Patrick Pirrotte, Cheryl Selinsky, Jeffrey R. Whiteaker, Haizhen Zhang, Jacob J. Kennedy, Chenwei Lin, Lik Wee Lee, Ping Yan, Nhan L. Tran, Landon J. Inge, Khaled Chalabi, Georges Decker, Rolf Bjerkvig, Amanda G. Paulovich, Guy Berchem, Yeoun Jin Kim

**Affiliations:** 1 Department of Oncology, Luxembourg Institute of Health, 1 A-B Rue Thomas Edison, L-1445 Strassen, Luxembourg; ksertamo@hotmail.com (K.S.); Rolf.Bjerkvig@uib.no (R.B.); berchem.guy@chl.lu (G.B.); yeounjin.kim@astrazeneca.com (Y.J.K.); 2 Competence Center for Methodology and Statistics, Luxembourg Institute of Health, 1 A-B Rue Thomas Edison, L-1445 Strassen, Luxembourg; Anna.Schritz@lih.lu; 3 Quantitative Biology Unit, Luxembourg Institute of Health, 1 A-B Rue Thomas Edison, L-1445 Strassen, Luxembourg; Sang-Yoon.Kim@lih.lu (S.-Y.K.); Antoine.Lesur@lih.lu (A.L.); Francois.Bernardin@lih.lu (F.B.); 4 Collaborative Center for Translational Mass Spectrometry, Translational Genomics Research Institute, 445 N Fifth St., Phoenix, AZ 85004, USA; nmo3@cdc.gov (K.P.); ppirrotte@tgen.org (P.P.); cselinsky@parkerici.org (C.S.); 5 Fred Hutchinson Cancer Research Center, 1100 Fairview Ave. N., Seattle, WA 98109-1024, USA; jwhiteak@fredhutch.org (J.R.W.); harryzhangdata@gmail.com (H.Z.); jkennedy@fredhutch.org (J.J.K.); clin@fredhutch.org (C.L.); leelikwe@gmail.com (L.W.L.); Yanping@gmail.com (P.Y.); apaulovi@fredhutch.org (A.G.P.); 6 Department of Cancer Biology, Mayo Clinic, 13400 E Shea Blvd, Scottsdale, AZ 85259, USA; Tran.Nhan@mayo.edu; 7 Norton Thoracic Institute, St. Joseph’s Hospital and Medical Center, Phoenix, AZ 85013, USA; landon.inge@roche.com; 8 Department of cardiac surgery, Institut national de chirurgie cardiaque et de cardiologie interventionnelle, 2A rue Nicolas-Ernest Barblé, L-1210 Luxembourg, Luxembourg; chalabi@incci.lu; 9 Zithaklinik, 46–48 rue d’Anvers, L-1130 Luxembourg, Luxembourg; georges.decker@hopitauxschuman.lu; 10 Department of Biomedicine, University of Bergen, Norway, Jonas Lies vei 91, N-5009 Bergen, Norway; 11 Centre Hospitalier de Luxembourg, 4 rue Nicolas-Ernest Barblé, L-1210 Luxembourg, Luxembourg

**Keywords:** lung cancer, plasma biomarker, targeted proteomics, parallel reaction monitoring, molecular diagnostics

## Abstract

Lung cancer is the deadliest cancer worldwide, mainly due to its advanced stage at the time of diagnosis. A non-invasive method for its early detection remains mandatory to improve patients’ survival. Plasma levels of 351 proteins were quantified by Liquid Chromatography-Parallel Reaction Monitoring (LC-PRM)-based mass spectrometry in 128 lung cancer patients and 93 healthy donors. Bootstrap sampling and least absolute shrinkage and selection operator (LASSO) penalization were used to find the best protein combination for outcome prediction. The PanelomiX platform was used to select the optimal biomarker thresholds. The panel was validated in 48 patients and 49 healthy volunteers. A 6-protein panel clearly distinguished lung cancer from healthy individuals. The panel displayed excellent performance: area under the receiver operating characteristic curve (AUC) = 0.999, positive predictive value (PPV) = 0.992, negative predictive value (NPV) = 0.989, specificity = 0.989 and sensitivity = 0.992. The panel detected lung cancer independently of the disease stage. The 6-protein panel and other sub-combinations displayed excellent results in the validation dataset. In conclusion, we identified a blood-based 6-protein panel as a diagnostic tool in lung cancer. Used as a routine test for high- and average-risk individuals, it may complement currently adopted techniques in lung cancer screening.

## 1. Introduction

Identifying solid cancers by a simple blood analysis has been a long-standing goal in cancer research as the detection of cancer during the regular screening can offer the patients immediate treatment solutions. While blood-based early diagnostics for cancer still remains a challenge, several proteins circulating in the blood have been useful for monitoring treatment response and/or tumor recurrence [1]. So far, only prostate-specific antigen is routinely measured in blood for early diagnosis of cancer [2]. 

Recently, Cohen and colleagues published the results of CancerSeek, a blood test that assesses the presence of 8 protein markers and 1933 genetic alterations in cell-free DNA to diagnose common solid tumors [3]. While the results were promising, the utility of this assay to advance cancer management has not yet garnered widespread adoption [4]. The median sensitivity of CancerSeek in lung cancer was ~59%, the second lowest among the 8 cancer types investigated [3]. 

Lung cancer is the most common malignancy in terms of incidence and the deadliest cancer worldwide [5,6]. The high lung cancer mortality is mainly based on an advanced level of progression at the time of diagnosis. Thus, the 5-year survival rate drops significantly from 83% for stage IA to 6% for stage IV tumors [7]. Only 15% of newly diagnosed lung tumors are diagnosed at an early stage [8]. In this context, lung cancer screening using low-dose computerized tomography (LDCT) can reduce lung cancer-specific mortality by 20% compared to chest radiography [9]. However, the high percentage of false-positive results and the malignancy risk associated with cumulative radiation exposure are serious limitations of LDCT. Therefore, a non-invasive, highly sensitive and specific method for early detection of lung cancer is essential to improve prognosis and reduce potential overdiagnosis. 

Previously, we have performed multi-omics discovery studies to find 4254 proteins associated to lung cancer. They were further narrowed down to 559 proteins as biomarker candidates potentially detectable in human blood [10,11]. For high-throughput screening, we have applied mass spectrometry (MS)-based proteomics technology that has greatly impacted clinical biomarker studies [12,13,14] and published a pilot study showing the efficacy of targeted proteomics for biomarker verification using selected reaction monitoring [13], where 95 potential protein biomarkers were screened in plasma samples from non-small cell lung cancer patients. We expanded this screening to a total of 559 biomarker candidates and concluded that 323 of them are detectable in human plasma by mass spectrometry. In the presenting study, we sought to develop a panel of proteins that clearly distinguishes lung cancer patients from healthy donors in plasma samples. Hence, we screened 351 proteins consisting of the 323 aforementioned biomarker candidates plus 28 additional plasma proteins [15], using parallel reaction monitoring (PRM), which dramatically increases the measurement specificity and simplifies the assay development [16]. A biomarker panel consisting of six proteins was identified with an outstanding sensitivity in distinguishing lung cancer patients from healthy individuals.

## 2. Results

### 2.1. Patient and Healthy Donor Demographics

The cohort was composed of 57.92% male and 42.08% female, with 14.93% non-smokers, 57.92% former smokers and 27.15% current smokers. The mean age was 63.56 (±10.03 standard deviation (SD)) and the median age was 63 (Table S1). No significant differences in age, gender and smoking status were found between healthy and cancer individuals.

### 2.2. Broad Selection of Potential Tumor Predictors in Plasma

Previous multi-omics discovery efforts performed in our laboratories suggested 559 proteins to be associated with lung cancer and potentially detectable in human blood (see Text S1: Discovery study summary) [10,11,17,18]. The detectability of each protein in human plasma was previously verified [13] resulting in a set of 323 proteins to be further verified in a larger cohort. In this study, the plasma levels of the 323 proteins were quantified by LC-PRM in plasma from lung cancer patients and healthy donors. An additional 28 well-known plasma proteins were also screened [15]. The list of the 351 proteins is shown in Table S2. Differential analysis of the PRM data indicated that plasma levels of 229 proteins were significantly different between lung cancer and healthy groups (Table S3).

### 2.3. Pathways Analysis and Interaction Network of the Differentially Expressed Proteins

**Figure 1:**
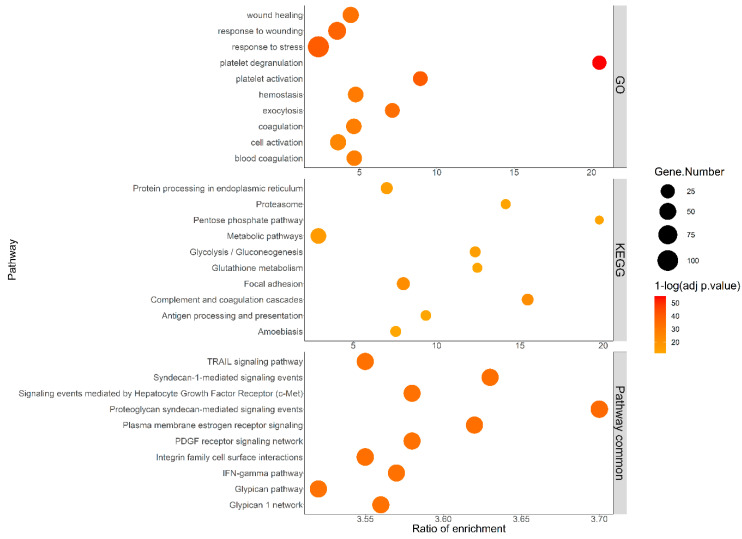
Pathway enrichment analysis of the differentially expressed proteins in plasma from lung cancer patients and healthy donors. The enrichment analysis was done using Pathway Commons, Kyoto encyclopedia of genes and genomes (KEGG) and gene ontology (GO) databases. The top 10 significantly enriched pathways are shown. The analysis was done based on the concentrations of the 229 differentially expressed proteins in plasma from lung cancer patients (*n* = 128) and healthy volunteers (*n* = 93).

In silico pathway analysis was performed to investigate whether the 229 differential proteins could cluster into functional pathways. As shown in Figure 1, proteoglycan (syndecan and glypican) and integrin networks were among the top 10 significantly enriched pathways, according to Pathway Commons. These networks are actively involved in tumor extracellular matrix (ECM) remodeling and cell–matrix interaction [19,20]. Signaling events mediated by estrogen receptor, hepatocyte growth factor (HGF) and platelet-derived growth factor (PDGF) receptors, all known to drive tumor growth [21,22,23], were also enriched. Importantly, the enrichment of interferon-gamma (IFN-γ) and tumor necrosis factor (TNF)-related apoptosis-inducing ligand (TRAIL) pathways may reflect the host immune responses towards the presence of the tumor. Additionally, plasma proteins were enriched in pathways related to glucose and fatty acid metabolism, alterations that are commonly observed in cancer. Gene ontology (GO) analysis reported “wound healing” and “response to wounding” as enriched pathways, probably due to a putative resemblance between ECM remodeling and ECM produced during wound healing [19].

The protein interaction network shown in Figure S1 describes the connection observed between the differentially expressed proteins. The epidermal growth factor receptor (EGFR), a known lung cancer driver, and the growth factor receptor-bound protein 2 (GRB2), an adaptor protein involved in many oncogenic signaling pathways appeared as main hubs in this network [24].

### 2.4. Refinement of Biomarker Selection

From the 229 differentially abundant proteins in plasma from lung cancer and healthy subjects, 90 proteins showed a correlation ≥ 0.9 or ≤ −0.9 with one or more proteins, whereas 139 proteins displayed weaker correlations. The dendrogram of correlations is shown in Figure S2. When a threshold of dissimilarity or “distance” between proteins was set to 0.1 (as an absolute value), 19 groups with highly correlated proteins were identified. Accordingly, 19 surrogate proteins were chosen (see the Materials and Methods Section for details) and 71 proteins were excluded from further analysis.

**Figure 2:**
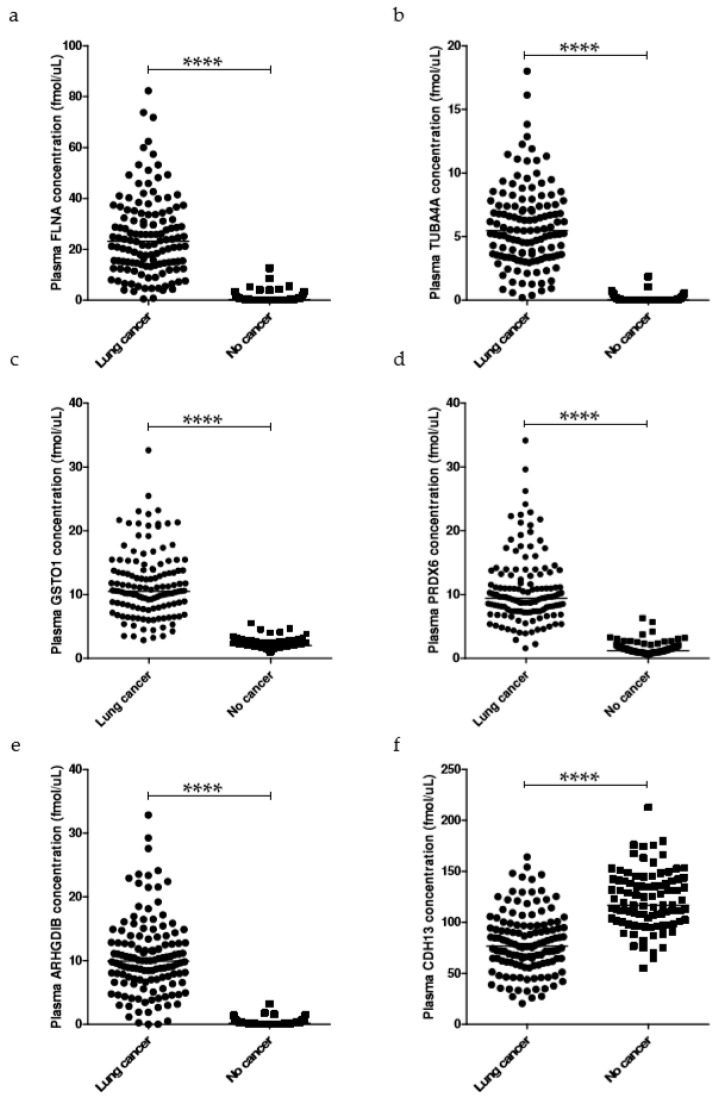
Plasma levels of the 6 protein biomarkers identified as a lung cancer diagnostic panel. Scatter plots of (**a**) filamin-A (FLNA), (**b**) tubulin alpha-4A chain (TUBA4A), (**c**) glutathione S-transferase omega-1 (GSTO1), (**d**) peroxiredoxin-6 (PRDX6), (**e**) rho GDP-dissociation inhibitor 2 (ARHGDIB) and (**f**) cadherin-13 (CDH13) concentrations obtained from lung cancer patients (*n* = 128) and healthy volunteers (*n* = 93) using the LC-PRM assay targeting proteotypic peptides. Data points and their median are shown. **** Adjusted *p* < 0.0001 using the non-parametric Kruskal–Wallis test.

Least absolute shrinkage and selection operator (LASSO) variable selection was implemented with 158 proteins. The combination that was retained the most (23 times) was filamin-A (FLNA), tubulin alpha-4A chain (TUBA4A), glutathione S-transferase omega-1 (GSTO1), peroxiredoxin-6 (PRDX6), rho GDP-dissociation inhibitor 2 (ARHGDIB) and cadherin-13 (CDH13) (hereafter referred to as 6-protein combination/panel/classifier) (Table S4). The concentrations of the 6 proteins were significantly different in plasma from lung cancer patients and healthy donors (Figure 2). The PRM readouts of the proteins measured in samples from one lung cancer patient and one healthy donor, compared to the internal standards, are shown in Figure S3. These proteins were individually selected as the most predictive ones, independently of the combination, in 74.51% of the cases for FLNA, 76.91% for TUBA4A, 44.42% for GSTO1, 54.74% for PRDX6, 45.11% for ARHGDIB and 81.43% for CDH13 (Table S4). The proteins that were selected as predictive in more than 75% of all combinations were TUBA4A, tissue factor pathway inhibitor (TFPI) and CDH13 (hereafter referred to as 3-protein combination).

### 2.5. Performance Analysis of the Models

We compared the performance of the models towards the commercially available Xpresys^®^ Lung (XL) test (Biodesix, Boulder, CO, USA) that consists of five diagnostic proteins [25]. The values of the performance indicators were the best with the 6-protein combination compared to the 3-protein combination, XL panel and the univariable models (Table 1): the lowest Akaike Information Criterion (AIC = 30.876), the highest area under the receiver operating characteristic curve (AUC = 0.999) (shared with the 3-protein combination), the highest positive predictive value (PPV = 0.992), the highest negative predictive value (NPV = 0.989), the highest specificity (0.989) (shared with ARHGDIB) and the highest sensitivity (0.992). The use of TUBA4A, TFPI and CDH13 as a classifier showed a slightly higher AIC (31.402) and slightly lower PPV (0.984), NPV (0.968), specificity (0.978) and sensitivity (0.977) values. When considering FLNA, TUBA4A, GSTO1, PRDX6 and ARHGDIB as sole classifiers, the performance indicators also showed excellent predictive power. Only CDH13 and TFPI performed worse, but still with a good predictive power (AUC = 0.845 and 0.851, respectively). 

Compared to the 6-protein model, the logistic regression model derived using the proteins of the XL panel had a higher AIC (45.592), suggesting a worse fit to the data. However, the AUC was very high (0.9962) and the PPV, NPV, specificity and sensitivity were only slightly lower than the ones of the 6-protein and 3-protein models (Table 1). We did not detect any statistically significant differences between AUC, sensitivities or specificities of the XL panel and the 6-protein combination.

Table S5 shows the lung cancer prediction of the 6-protein panel in the different subject groups of the training cohort, classified by cancer type, stage, grade and by smoking history. One patient with stage I adenocarcinoma (ADC), a current smoker, was falsely predicted as not having lung cancer. Another current smoker was falsely classified as having lung cancer. As the number of cases per category are quite small, no clear statement can be made on whether a specific cancer type, stage, grade or smoking history of a subject is influencing the predictive ability of the biomarker panel.

We then tested the ability of the 6-protein panel to predict cancer stage. As shown in Table 2, the 6-protein panel distinguished between healthy and lung cancer individuals but could not predict cancer stage. An unweighted Cohen’s Kappa of 0.59 (95% confidence interval (CI), 0.52–0.66) and a weighted Cohen’s Kappa of 0.73 (95% CI, 0.73–0.73) were found, suggesting a moderate degree of agreement between predicted and clinically annotated stages. Importantly, the 6-protein panel classified 22 out of 23 stage I patients as lung cancer individuals, demonstrating its strong diagnostic performance in early-stage cases.

### 2.6. Determination of Biomarker Thresholds for Outcome Prediction

We used the PanelomiX platform to select the best thresholds for the 6 biomarkers identified. Three panel optimization options were used: optimizing the sensitivity at ≥95% specificity, optimizing the specificity at ≥95% sensitivity and optimizing global accuracy. When choosing to optimize the accuracy or the specificity, only one threshold per biomarker was selected by Panelomix, resulting in one combination per optimization. When optimizing the sensitivity, 19,644 combinations were found, with the first one being the same as the threshold combination selected when optimizing the specificity. Therefore, two threshold combinations were considered: the one obtained when optimizing the panel accuracy (T_A_ combination) and the combination common to sensitivity and specificity optimization (T_S_ combination) (Table 3). If any 3 proteins were positive using T_A_ thresholds, then the subject was classified as having lung cancer. For T_S_, any 5 of the 6 proteins have to be positive in order to classify an individual as having lung cancer. 

Applying the thresholds on the original dataset, the performance metrics of the panel were excellent: a sensitivity of 0.992 and a specificity of 0.989 for T_A_ combination, and a sensitivity of 0.977 and a specificity of 1.0 for T_S_ combination.

### 2.7. Panel Performance on the Validation Dataset

The models were then tested on a validation dataset using plasma from 48 lung cancer patients and 49 healthy donors. The models’ estimates of the logistic regression and Panelomix thresholds obtained from the training set were applied to the validation set for cancer prediction. NPV, PPV, sensitivity, specificity and AUC of the XL and the 6-protein panels were calculated for the new dataset (Table 4). When comparing the results obtained from the logistic regression models, values of all the performance metrics of the 6-protein combination were at least as high as the values of the XL panel. Interestingly, the highest specificity (0.918) was obtained for the 6-protein panel, as predicted by the T_S_ thresholds. All the possible sub-combinations of the 6-protein panel were also tested on the validation dataset. Many of them displayed excellent performance, as shown by the forest plots of NPV, PPV, sensitivity, specificity and AUC (Figures S4–S8). Table S6 shows the lung cancer prediction in the different subgroups classified by cancer type, stage, grade and by smoking history. One patient with stage I ADC and two large cell carcinoma (LCC) patients (stage I and stage III), both having grade III tumors, were falsely classified as being lung cancer-negative. The 3 patients were former smokers. Among the healthy subjects, 6 were falsely classified as having lung cancer: 1 current smoker, 4 former smokers and 1 subject who never smoked. As stated before, due the small number of cases, it is not possible to conclude whether a specific cancer type, stage, grade or smoking history affects the predictive ability of the 6-protein panel.

## 3. Discussion

At present, more than half of lung cancer patients are diagnosed at a metastatic stage [26]. Early diagnosis is a prerequisite for improved patient survival and treatment outcome. When compared to chest radiography, the use of LDCT for lung cancer screening clearly demonstrated a mortality benefit [9,27]. However, several issues are associated with imaging techniques, mainly the high percentage of false-positive results (96.4% and 94.5% in the LDCT and the radiography groups, respectively) [9]. If combined with a highly accurate measurement method, blood samples may represent an ideal minimally invasive, easily collected material for cancer diagnostics.

The purpose of this study was to identify a panel of protein biomarkers to be used as a non-invasive diagnostic tool in lung cancer. For this purpose, 351 potential biomarkers were screened, that have been discovered and preliminarily verified in human plasma [13]. Here, based on PRM measurement followed by logistic regression analysis, we identified a blood-based 6-protein panel as a potential diagnostic tool in lung cancer. In order to make this panel easy to use by medical practitioners, we also adopted a threshold-based approach, attributing a cut-off value per biomarker, then a score per sample to classify it as lung cancer or healthy.

The biomarker panel displayed excellent performance in the test cohort, supported by the AUC (0.999), PPV (0.992), NPV (0.989), specificity (0.989) and sensitivity (0.992) values. The results were confirmed in a validation dataset which also showed that other sub-combinations of these 6 proteins displayed excellent discriminative power. Importantly, the 6-protein panel non-invasively detected lung cancer at different stages of the disease (including stage I), suggesting its high potential as a screening tool.

The performance of our biomarker panel was compared to a commercially available, MS-based lung cancer diagnostic test, Xpresys^®^ Lung (XL) test. The XL test is a multiprotein plasma classifier consisting of five diagnostic plasma proteins, originally designed to differentiate benign from malignant lung nodules among indeterminate pulmonary nodules [25,28,29]. While limited, based on different primary objectives and target populations, direct comparison with the XL test in the same pool of plasma samples can provide a useful benchmark for our panel. In the training set, the values of all performance metrics tended to be better with our 6-protein panel; however, the differences were not statistically significant, suggesting that both panels displayed a good diagnostic accuracy in our cohort. 

The origin of circulating proteins differs from molecule to molecule. For example, the outer parts of membrane proteins overly expressed on cancer cells can be shed into body fluids, as in the case of the detectable serum human epidermal growth factor receptor 2 (HER2) in breast cancer patients [30,31]. The invasive cancer cell structure can disrupt tissue architecture and creates gaps between cellular compartments, leading to a leak of interstitial fluids into the circulation, as in the case of high prostate-specific antigen (PSA) serum level in prostate cancer patients [32]. An elevated level of a protein in the blood can result from its increased secretion from the diseased tissue (e.g., alpha-fetoprotein in liver cancer) [33] or it can be caused by the inflammation associated with cancer (e.g., increased production of serum amyloid A in lung cancer patients) [34]. Any of these proteins, or more likely a combination of them, could be used as tumor markers if they are detectable and specific to cancer.

Here, we showed that the differentially abundant proteins in plasma from lung cancer and healthy subjects were mainly involved in pathways associated with tumor growth, ECM remodeling, invasion and immune responses. Only 55 proteins of the 229 candidate biomarkers were reported as secreted in the Uniprot database. However, all of the 229 proteins were previously identified in extracellular vesicles or in exosomes (according to Vesiclepedia and Exocarta), suggesting that they may be shed by cells and released into the blood via plasma vesicles. Our data strongly suggest that the changes observed in the plasma proteome from lung cancer patients may be derived not only from the tumor itself but also from the tumor microenvironment and host tissues. Our findings are thus in line with previous proteomics data obtained in plasma from a mouse model of mammary cancer [35].

The 6-protein diagnostic panel consisted of FLNA, PRDX6 and ARHGDIB, associated with tumor growth, cell invasion and metastasis [36,37,38,39,40,41], GSTO1, having an antioxidant defense role (together with PRDX6) [38,41,42,43], TUBA4A, found enriched in serum exosomes from NSCLC patients [44], and the tumor suppressor CDH13 [45]. The increased levels of GSTO1 and PRDX6 in plasma of lung cancer patients may reflect their protective role in the cancer redox environment, or may be associated with the activation of antioxidant pathways resulting from cigarette smoking. Interestingly, IDH1, which also plays a protective role in the tumor-associated redox process, has been recently proposed as a promising plasma biomarker for the diagnosis of lung ADC [46].

The cohort used in this study consisted of patients from different lung cancer stages and a majority of healthy donors with a smoking history, similarly to the intended use population for this blood-based classifier. Therefore, the obtained high NPV cannot be due to the low prevalence of lung cancer, which is of 58% in our study cohort (128 lung cancer patients and 93 healthy subjects). However, since the PPV increases with the incidence rate of the disease, the panel’s accuracy has yet to be demonstrated in the appropriate screening population, where the lung cancer incidence rate is about 53.5 and 47.6 per 100,000, among men and women, respectively [47].

Our discovery phase studies have carefully selected 559 candidate proteins with a strong pre-screening evidence including serum analysis of xenograft mouse models. This led to the high success rate (41%) of candidate biomarkers at the high-throughput PRM screening. However, all the clinical samples used in this study were collected and processed by one organization, which may introduce an unknown bias. External validation on several datasets obtained from a wide range of samples collected, processed and analyzed by different investigators from different centers will help to randomize potential bias, and thus reduce false discovery. Limitations of this study include its inability to demonstrate that the biomarker panel is detecting only lung cancer among other malignancies, and that the results are not due to other lung conditions commonly associated with lung cancer, such as chronic obstructive pulmonary disease [4]. Therefore, the panel needs to be validated in independent cohorts including patients with different cancer types and donors with and without underlying non-malignant lung diseases to precisely estimate its diagnostic power.

Since we concluded a defined set of six proteins, now we can modify the LC-PRM method to perform much faster quantitative analysis. In this present study, we used three multiplexed (117 targets per each method) PRM with 66-min LC separation, resulting in a total of 3.3-h separation time to screen 351 targets. The LC separation time can be reduced to less than 5 min for six targets with increased datapoints and higher mass resolution. This will increase both throughput and assay sensitivity, and thus allow us to expand the sample size including various clinical status required for further validation.

## 4. Materials and Methods 

### 4.1. Study Cohort

The training cohort consisted of 128 lung cancer patients and 93 healthy donors followed within Luxembourg’s hospitals. The validation cohort comprised 48 patients and 49 age, sex and smoking status-matched non-cancer subjects, not included in the training cohort. All the participants provided blood samples following informed consent according to the Helsinki Declaration. The study was approved by the national research ethics committee “Comité National d’Ethique de Recherche” and the national commission for data protection “Commission Nationale pour la Protection des Données”. Blood samples were collected and processed following the standard operating procedures of the Integrated Biobank of Luxembourg to prepare plasma samples. Diagnosis, staging and grading of the disease were done by experienced pathologists, according to the IASLC/ATS/ERS histological classification of lung tumors (2011) and TNM classification of lung carcinoma (2009) [48,49]. The clinicopathological features of the subjects are summarized in Tables S7 and S8.

### 4.2. Plasma Depletion and Processing

For the training cohort, high abundance proteins were removed from 40 µL of plasma using an Agilent 1260 Infinity Bio-inert LC system equipped with a Human 14 Multiple Affinity Removal Column (4.6 × 100 mm) (Agilent Technologies, Diegem, Belgium) according to the manufacturer’s procedure. After elution, buffer A was exchanged to 100 mM NH_4_HCO_3_/10% ACN (pH 8) and the volume was reduced to 100 µL using a spin concentrator 5K (Agilent). Proteins were denatured with 1% sodium deoxycholate (SDC), reduced with 10 mM dithiothreitol for 30 min at 37 °C, alkylated with 25 mM iodoacetamide for 30 min at room temperature, followed by quenching with 10 mM n-acetyl-L-cysteine. All reagents were prepared in 50 mM tris buffer. The processed sample was diluted to reduce the SDC concentration to 0.5% and incubated with 13 µg of sequencing grade trypsin (Promega, Leiden, The Netherlands) for 16 h at 37 °C, then with 10 U of PNGase F for 1 h at 37 °C followed by an additional 2 µg of trypsin for 3 h at 37 °C. SDC was removed by precipitation with 1% formic acid and centrifugation. Digested samples were cleaned up with Sep-Pak C18 cartridges (Waters, Milford, MA, USA) and dried in vacuo. Samples were reconstituted with 200 µL of 0.1% formic acid/4% acetonitrile. Minor modifications to the protocol were made for the samples of the validation cohort (Text S2: Supplementary Materials and Methods).

### 4.3. LC-PRM Analysis

Stable isotope labeled (SIL) (^13^C_6_^15^N_4_ for the C-terminal arginine and ^13^C_6_^15^N_2_ for the C-terminal lysine) synthetic peptides were used as internal standards (AQUA QuantPro grade, Thermo Fisher Scientific, Bremen, Germany). For each peptide, LC-MS attributes (retention time, precursor m/z and the most intense fragment ions) were determined to build the LC-PRM method. Samples were analyzed using scheduled LC-PRM assays for 351 peptides (Table S2). An Ultimate 3000 RSLCnano system coupled to a Q-Exactive Plus mass spectrometer (Thermo Fisher Scientific) was used as described previously [16]. Precise, relative quantification was obtained from the intensity ratio of light and SIL peptides. Details of LC-MS and data processing generated with the validation cohort are available in Text S2: Supplementary Materials and Methods.

### 4.4. Model Development and Statistical Analysis

The LC-PRM signal was converted into plasma protein concentration in fmol/µL based on the internal standard peptides. Values of undetected proteins were replaced by minimal protein concentration/√2. Non-parametric Kruskal–Wallis test and Bonferroni adjusted *p*-values were used to compare protein concentrations in lung cancer and healthy samples. Proteins with *p*-value < 0.00014 (= 0.05/351; Bonferroni corrected) were further considered for analysis. Correlations between proteins were investigated using Spearman’s correlation coefficient. Hierarchical clustering of proteins was performed using a dissimilarity function (= 1 − absolute value of correlation) to discriminate all correlated groups. One protein per group of highly correlated proteins was selected to represent the group, based on high intensity, lower missing values in lung cancer samples and absence of interference in PRM signals. 

Bootstrap sampling and least absolute shrinkage and selection operator (LASSO) penalization were used to find the best combination of proteins for outcome prediction. LASSO with 10-fold cross-validation was performed on 4,500,000 bootstrapped datasets, using the “glmnet” package of R. To assess the predictive power of proteins and protein combinations, the NPV, PPV, sensitivity, specificity, AUC and AIC of the logistic regression models were calculated on the original dataset. A bootstrap test was used to compare AUC of different models. For comparing sensitivities and specificities, the McNemar χ^2^ test was used, as recommended [50]. For model validation, sensitivity, specificity, NPV, PPV, AUC and their 95% CI were calculated on the validation dataset.

Multinomial logistic regression was used to predict the probability of each cancer stage (6 levels, including 4 cancer stages, 1 unknown stage and 1 healthy condition) using the 6-protein panel. The level with the highest probability was chosen as the final predicted cancer stage (or healthy condition). The Cohen’s kappa test was used to evaluate the degree of agreement between clinically annotated and predicted staging [51]. 

Continuous variables were compared using the Kruskal–Wallis test. Binary or categorical variables were compared using Pearson’s Chi-Squared test.

### 4.5. Use of PanelomiX for Threshold Selection

The PanelomiX platform was used to select thresholds for the candidate biomarkers to have the optimal classification performance of the combination [52]. First, a threshold value was defined for each of the proteins, then a score was assigned to each subject. A patient’s score is the number of biomarkers fulfilling the disease condition (referred to as “positive” biomarker). A subject was classified as a lung cancer patient if their score was at least equal to a panel threshold score identified by Panelomix. Thresholds obtained from the training set were applied to the validation set for cancer prediction and the performance metrics were calculated. 

### 4.6. Pathway Analysis and Protein Interaction

The enrichment analysis was done using Pathway Commons, KEGG and GO databases and “hsapiens_entrezgene_protein-coding” as a reference set. The statistical evaluation of the enrichment was performed using the hypergeometric test and the method of Benjamini and Hochberg for *p*-value adjustment. A pathway was considered significantly enriched if the *p*-value was <0.05 and if it contained at least 2 genes from the query list. The Functional Enrichment Analysis tool “FunRich” was used to visualize protein–protein interactions. 

## 5. Conclusions

In this study we identified a protein-based diagnostic panel to detect lung cancer using a non-invasive material (blood), a non-radiative, highly sensitive and highly specific method. If used as a routine test for high- and average-risk individuals (e.g., smokers and former smokers), it may efficiently complement LDCT in lung cancer screening. This would reduce the number of false-positive cases that often lead to additional invasive tests and unnecessary costs and expose the patients to physical and mental hardships.

## Figures and Tables

**Table 1:** Performance of the logistic regression models in tumor prediction.

Model	AIC	AUC	PPV	NPV	Specificity	Sensitivity
6-protein combination	30.876	0.999	0.992	0.989	0.989	0.992
3-protein combination	31.402	0.999	0.984	0.968	0.978	0.977
FLNA	65.647	0.990	0.967	0.908	0.957	0.930
TUBA4A	41.556	0.997	0.984	0.948	0.978	0.961
GSTO1	45.427	0.996	0.976	0.947	0.968	0.961
PRDX6	51.763	0.993	0.976	0.957	0.968	0.969
ARHGDIB	54.303	0.981	0.992	0.929	0.989	0.945
CDH13	219.090	0.845	0.791	0.747	0.699	0.828
TFPI	204.860	0.851	0.836	0.737	0.785	0.797
Xpresys^®^ XL panel	45.592	0.996	0.969	0.957	0.957	0.969
ALDOA	43.946	0.994	0.969	0.947	0.957	0.961
COL18A1	250.790	0.767	0.752	0.630	0.677	0.711
FTL	297.720	0.554	0.579	NaN	0.000	1.000
LGALS3BP	295.220	0.601	0.601	0.500	0.258	0.813
THBS1	161.780	0.924	0.871	0.794	0.828	0.844

FLNA = Filamin-A; TUBA4A = Tubulin alpha-4A chain; GSTO1 = Glutathione S-transferase omega-1; PRDX6 = Peroxiredoxin-6; ARHGDIB = Rho GDP-dissociation inhibitor 2; CDH13 = Cadherin-13; TFPI = Tissue factor pathway inhibitor; ALDOA = Fructose-bisphosphate aldolase A; COL18A1 = Collagen alpha-1(XVIII) chain; FTL = Ferritin light chain; LGALS3BP = Galectin-3-binding protein; THBS1 = Thrombospondin-1; AIC = Akaike Information Criterion; AUC = Area under the receiver operating characteristic curve; PPV = Positive predictive value; NPV = Negative predictive value; NaN = Not a number (cannot be calculated since no patient was classified as not having a cancer).

**Table 2:** Number of clinically annotated and predicted healthy and lung cancer patients, including their stages, as obtained using the 6-protein classifier.

Cancer stages			Clinically Annotated Stages				
		No cancer	Stage NA *	Stage I	Stage II	Stage III	Stage IV
	**No cancer**	92	1	1	0	1	0
**Predicted stages**	Stage NA *	0	2	0	1	0	0
	**Stage I**	0	2	9	1	2	6
	**Stage II**	0	0	0	0	0	1
	**Stage III**	0	0	0	1	0	0
	**Stage IV**	1	6	13	8	16	57
	**Sum**	93	11	23	11	19	64

* NA = not available.

**Table 3:** Threshold values and positivity of the biomarkers when optimizing the global accuracy (T_A_) or the sensitivity or specificity (T_S_) of the panel, as defined by PanelomiX platform.

Protein Biomarker	T_A_	T_S_
FLNA	>0.48091298	>0.48091298
TUBA4A	>1.6875327	>0.18983749
GSTO1	>5.363042	>5.363042
PRDX6	>5.9975386	>4.038682
ARHGDIB	>0.5091874	>0.5091874
CDH13	<69.826614	<148.1571

**Table 4:** Performance of the classification models on the validation dataset.

Performance metrics	6-Protein Panel			Xpresys^®^ XL Panel
	T_A_ Thresholds	T_S_ Thresholds	Logistic Regression	Logistic Regression
NPV (95% CI)	0.840 (0.709–0.928)	0.849 (0.724–0.933)	0.935 (0.821–0.986)	0.930 (0.809–0.985)
PPV (95% CI)	0.851 (0.717–0.938)	0.909 (0.783–0.975)	0.882 (0.761–0.956)	0.833 (0.707–0.921)
Sensitivity (95% CI)	0.833 (0.698–0.925)	0.833 (0.698–0.925)	0.938 (0.828–0.987)	0.938 (0.828–0.987)
Specificity (95% CI)	0.857 (0.728–0.941)	0.918 (0.804–0.977)	0.878 (0.752–0.954)	0.816 (0.680–0.912)
AUC (95% CI)	0.845 (0.773–0.918)	0.876 (0.810–0.942)	0.908 (0.850–0.965)	0.877 (0.812–0.942)

## Supplementary Materials

The following are available online at https://www.mdpi.com/2072-6694/12/6/1629/s1: Figure S1: Interaction network of differentially expressed proteins in the plasma of lung cancer patients versus healthy donors, Figure S2: Dendrogram of correlation «distances», Figure S3: PRM readouts of the 6 proteins included in the diagnostic panel, Figure S4: Forest plot showing the NPV values of all the possible sub-combinations of the 6-protein panel, Figure S5: Forest plot showing the PPV values of all the possible sub-combinations of the 6-protein panel, Figure S6: Forest plot showing the sensitivity values of all the possible sub-combinations of the 6-protein panel, Figure S7: Forest plot showing the specificity values of all the possible sub-combinations of the 6-protein panel, Figure S8: Forest plot showing the AUC values of all the possible sub-combinations of the 6-protein panel, Table S1: Patient and healthy donor demographics, Table S2: List of target proteins consisting of 323 potential biomarker candidates (in bold) and 28 typical plasma proteins, and their surrogate peptides and precursors m/z, Table S3: List of the 229 proteins differentially expressed in plasma from lung cancer patients and healthy donors, as measured by LC-PRM, Table S4: Protein combinations selected more than 10 times in LASSO as the most predictive ones in distinguishing lung cancer from healthy samples, and the percentage of appearance of individual proteins in 4,500,000 bootstrapped datasets, Table S5: The lung cancer prediction of the 6-protein panel in the training cohort classified by lung cancer type, stage, grade and by smoking history, Table S6: The lung cancer prediction of the 6-protein panel in the validation cohort classified by lung cancer type, stage, grade and by smoking history, Table S7: Clinicopathological features of lung cancer patients in the training (T) and in the validation (V) cohorts, Table S8: Clinicopathological features of healthy donors in the training (T) and in the validation (V) cohorts, Text S1: Discovery study summary, Text S2: Supplementary Materials and Methods.
